# Yoga, Dance, Team Sports, or Individual Sports: Does the Type of Exercise Matter? An Online Study Investigating the Relationships Between Different Types of Exercise, Body Image, and Well-Being in Regular Exercise Practitioners

**DOI:** 10.3389/fpsyg.2021.621272

**Published:** 2021-03-24

**Authors:** Verena Marschin, Cornelia Herbert

**Affiliations:** Applied Emotion and Motivation Psychology, Institute of Psychology and Education, Ulm University, Ulm, Germany

**Keywords:** body image, physical activity, exercise, ballroom dance, yoga, physical efficacy, well-being

## Abstract

Physical activity, specifically exercising, has been suggested to improve body image, mental health, and well-being. With respect to body image, previous findings highlight a general benefit of exercise. This study investigates whether the relationship between exercising and body image varies with the type of exercise that individuals preferentially and regularly engage in. In addition, physical efficacy was explored as a potential psychological mediator between type of exercise and body image. Using a cross-sectional design, healthy regular exercise practitioners of yoga, ballroom dance, team sports, or individual sports as well as healthy adults reporting no regular exercising were surveyed. Body image and its different facets were assessed by a set of standardized self-report questionnaires, covering perceptual, cognitive, and affective body image dimensions particularly related to negative body image. In addition, participants were questioned with regard to mental health. Participants were 270 healthy adults. Descriptive statistics, measures of variance (ANOVA), and multiple linear regression analysis with orthogonal contrasts were performed to investigate differences between the different exercise and non-exercise groups in the variables of interest. In line with the hypotheses and previous findings, the statistic comparisons revealed that body dissatisfaction (as one important factor of negative body image) was most pronounced in the non-exercise group compared to all exercise groups [contrast: no exercise versus exercise (all groups taken together)]. Physical efficacy, as assessed with a standardized questionnaire, mediated the difference between type of exercise (using contrasts) and body image including perceptual, cognitive, and affective body image dimensions. The findings shed light on so far less systematically investigated questions regarding the relationship between types of exercise, like yoga and ballroom dance, and body image. The results underscore the relevance of considering possible influencing factors in exercise research, such as the perception of one’s physical efficacy as a mediator of this relationship.

## Introduction

It is generally agreed upon that physical activity is associated with numerous health benefits. In several studies physical activity was found to be associated with health-related advantages. Among them are faster heart rate recovery from acute stressors, lower blood pressure at rest, weight loss, and lower risk of cardiovascular and non-communicable chronic diseases (Cornelissen and Fagard, 2005; Penedo and Dahn, 2005; Fagard, 2006; Forcier et al., 2006; Blair and Morris, 2009). In order to promote and maintain physical health, the World Health Organization (World Health Organization [WHO], 2010) advises adults aged 18 to 64 years to carry out 150 min of moderate-intensity physical activity (e.g., housework or dancing) per week. Alternatively, 75 min of vigorous-intensity physical activity (e.g., running) per week, or of a comparable combination of moderate- and vigorous-intensity exercise, are recommended (World Health Organization [WHO], 2010). On top of that, aerobic exercise as well as muscle-strengthening activities are advised. The term “exercise” needs to be differentiated from physical activity as a structured and planned form of physical activity (Caspersen et al., 1985; Budde et al., 2016). Up to 25% of the world’s population do not meet these physical activity recommendations, and levels of physical inactivity are increasing (World Health Organization [WHO], 2010; Du et al., 2019). Physical inactivity has many negative physical and mental consequences. It adversely affects the cardiovascular system, the central nervous system, bone density, immunity, quality of life, anxiety, depression, and other important health factors (Booth et al., 2017). The mortality risk of physically inactive (not meeting the recommendations) versus active people is up to 20 to 30% higher (World Health Organization [WHO], 2010).

It can be expected that there are differences in health outcomes in dependence of the type of exercise carried out. Most of the studies so far focused on health improvements associated with aerobic exercise where the aerobic metabolism provides oxygen in order to meet energy demands (McArdle et al., 2006; Mikkelsen et al., 2017). Alternative types of exercise, e.g., yoga or dancing, are so far not sufficiently evaluated. These types of exercise can also have an impact on the cardiovascular system. Additionally, they focus on muscular strength, coordination, flexibility, balance, and relaxation (Mikkelsen et al., 2017). Regarding the general population, ballroom dancing and yoga have become particularly famous over the past few years as leisure time activity as well as exercise intervention for fostering and maintaining a healthy lifestyle. Moreover, exercises such as yoga and dancing can improve mental health and well-being, as regular physical activity with a focus on endurance exercises, such as jogging, cycling, or swimming, do.

Not much is known scientifically about the consequences of ballroom dance on physical and mental health and well-being. Ballroom dance is associated with enhanced body composition, improved balance, gait, and flexibility (Fong Yan et al., 2018). Furthermore, compared to other exercises, improvements in cardiovascular function, such as lower resting heart rate and better heart rate recovery, were found in cross-sectional studies comparing male practitioners of recreational ballroom dancing with a reference group of physically non-active individuals (Cruz et al., 2017). Mental health may also be positively affected. In a study by Koch et al. (2007), depressive patients had significantly lower depression scores and higher vitality after having participated in a dance intervention compared to a control group of depressive participants having listened to music or having taken part in ergometer exercise. Ballroom dance is a social dance in which body movements and their synchronization between partners play a prominent role (Cohen-Stratyner, 2020). Because ballroom dance steps can be adjusted in pace and steps and because ballroom dance improves body posture and body expression, it can also be performed by people with physical constraints and by different age groups (Hackney and Earhart, 2010; Vankova et al., 2014). Ballroom dance can be classified as aesthetic type of exercise (Fong Yan et al., 2018). Aesthetic exercises set the focus on the physical appearance of the body. In most aesthetic types of exercise, e.g., gymnastics or ballet dance, evaluation of one’s own proficiency is to a large extent based on looks and expression. Still, if ballroom dance is carried out in a non-competitive way, as it is taught in most regular dance schools, aesthetic and appearance are of less relevance and less focused on. Both gymnastics and ballet dance include stages of evaluation. Evaluation in ballroom dance if practiced as a recreational activity usually only takes place if couples want to get individual feedback on their dancing. Therefore, it can be expected that the focus in ballroom dance lies merely on the appearance of the steps and movements instead of the appearance of the body. As far as yoga is concerned, different effects of yoga on physical and mental health have been examined during the last few years. Increased physical well-being (e.g., physical flexibility, spine mobility, muscle endurance, cardiovascular activity), improved cognitive functions and emotional well-being were reported for individuals practicing yoga in comparison to other exercise control groups. The correlation between yoga practice and psychological well-being might be best explained by a heightened body awareness and positive body image (Tihanyi et al., 2016).

### Body Image and Its Relationship With Exercise

Theoretically, body image has been defined as a multifaceted and multidimensional construct. As such, it encompasses several aspects of the bodily self that are related to perceptual, cognitive, affective, and behavioral facets of self-perception (Cash, 2004). Body image includes the person’s emotional attitudes, beliefs, and perceptions of the own body. On top of that, it comprises a person’s beliefs and feelings about one’s own appearance, the sense and control of the body during movement, and one’s own appraisal of body shape, including height and weight. Body dissatisfaction, i.e., negative attitudes toward one’s own body size including one’s physical appearance, as well as discrepancies between actual and ideal body size are cognitive, affective, and perceptual indicators of a negative body image. Thus, a negative body image manifests itself by how one sees oneself (perception) and by how one thinks and feels about the way one looks (cognitive, affective). Dissatisfaction with body size is measured as perceived discrepancy between the actual and desired ideal body including shape, size, height, or weight (Grogan, 2017). This discrepancy between the actual and ideal body describes the perceptual dimension of a negative body image: the larger the perceptual discrepancy, the more pronounced is the dissatisfaction with body size in terms of size, weight, and shape. In addition, it can be used as an index of body size distortion, i.e., the misjudgment of one’s own body shape and size (Gardner and Boice, 2004). Dissatisfaction with the own body in terms of size, weight, and shape is one of the main reasons for the maintenance of a negative body image and can be associated with adverse health consequences like eating behavior pathology, low self-esteem, psychological distress, and depressive symptomatology (Olivardia et al., 2004; Johnson and Wardle, 2005; Solomon-Krakus et al., 2017). Furthermore, cognitive and affective aspects of body dissatisfaction were found to be a predictor of dysfunctional exercise in adolescence, obesity, and physical inactivity (Voelker et al., 2015).

As suggested by meta-analytic studies, there exist a number of studies that investigated the relationships between body image, mental or physical health, and exercise (Hausenblas and Fallon, 2006; Campbell and Hausenblas, 2009). Several of these studies included in these meta-analyses used a diverse set of measures, scales, and questionnaires to investigate the relationship between exercise and body image. Most studies had a focus on body dissatisfaction as one of the important factors contributing to a negative body image. In summary, the meta-analytic results confirm that (a) exercise interventions can improve negative body image (Hausenblas and Fallon, 2006; Campbell and Hausenblas, 2009; Salci and Martin Ginis, 2017) and (b) exercisers differ in body image compared to non-exercisers (Hausenblas and Fallon, 2006). However, whether the relationship between exercise and body image, negative body image in particular, is comparable across varying types of exercise needs to be further explored. A study by Morano et al. (2011) for instance found that team sports practitioners (e.g., soccer players) do have a lower body dissatisfaction compared to individual sports practitioners (e.g., practitioners of martial arts such as karate). Differences in body dissatisfaction between practitioners of different types of exercise might be related to the relevance of body weight and the relative focus on leanness of the body which in turn might promote higher body dissatisfaction (Dyremyhr et al., 2014).

As far as different types of dance are concerned, a differential relationship with body image might be expected. Some types of dance (when not practiced at a professional level) focus more on the functionality of the body (e.g., ballroom dance could be an example) than on appearance. The focus lies more on the capability of the body and physical competence and empowerment, sensing what one’s own body can do instead of how it looks and how it is perceived by others (Tiggemann, 2015; Hill et al., 2016). Evidence for a relationship between ballroom dance and body image is however scarce. Ballroom dance comprises the perception of one’s own body movements and the perception of the movement and articulation of the body of the partner, even of other couples in the room (Fonseca et al., 2014). Therefore, it could be assumed that ballroom dance improves body image and has a positive effect on the bodily self (including body schema and body awareness). Whether ballroom dancing is however capable of reducing a negative body image has not been investigated in detail so far. In a previous study, practice of non-competitive ballroom dancing showed positive effects on body perception as measured with the projection point test, a tactile measure of body size perception (Fonseca et al., 2014). As pointed out by Swami and Harris (2012), the effects of dancing may additionally depend on dance type and level (years of practice, beginner versus advanced, etc.).

As far as yoga is concerned, in clinical practice yoga has already fully established as an intervention to improve negative body image and body dissatisfaction in women. Also, it has been successfully included in the treatment of body image distortions in eating disorders (EDs) and as intervention preventing EDs in studies investigating individuals at risk for EDs (Lauche et al., 2017). As far as the general population is concerned, there are results supporting the notion that yoga practitioners score higher on body appreciation measured via the Body Appreciation Scale-2 (Tylka and Wood-Barcalow, 2015) than non-yoga practitioners (Mahlo and Tiggemann, 2016), and better embodiment (own relationship with the body through which one can realize and respect one’s own needs and experiences of the body; Piran, 2015) was found to be a mediator. Practicing yoga has been shown to be perceived particularly by young adults as beneficial for the improvement of one’s body image irrespective of gender (Neumark-Sztainer et al., 2018; Ariel-Donges et al., 2019). Thus, current evidence suggests that regular exercise engagement might be associated with both positive and negative effects on body dissatisfaction and effects might vary with the type of exercise.

### Body Image and Exercise: Possible Mediating Factors

As is obvious from the above cited studies and their findings, several physical and psychological factors could exist on which exercisers and non-exercisers might differ from each other. However, only some psychological factors might be of particular relevance when investigating differences between practitioners of different types of exercise with respect to negative body image. One of these psychological factors that could act as mediator between type of exercise and negative body image is physical efficacy. Physical efficacy is defined as the attitude toward one’s body not only in terms of strength but also in terms of flexibility and agility. Physical efficacy has been suggested to be an important factor of one’s bodily self-concept (Deusinger, 1998). As such, it focuses on a person’s subjective perception of his/her physical strength and physical skills in general, unrelated to a certain task or behavior. Studies emphasize the importance of the subjective aspect associated with the concept of physical efficacy, namely the perception of one’s physical strength and skills rather than the actual physical skills of the person (Salci and Martin Ginis, 2017; Peers et al., 2020). Self-perception of one’s physical strength and skills was found to be a significant mediator responsible for positive effects of exercise on body image in children (Peers et al., 2020) and in women with pre-existing body image concerns after an acute bout of exercise (Salci and Martin Ginis, 2017). Whether physical efficacy might mediate differences between exercise and body image in different types of exercise in adults remains to be explored.

### Aim of the Current Study

Against this background of scientific evidence, the central questions that motivated the present study were to investigate (a) whether the relationship between exercising and body image varies with the type of exercise that individuals preferentially and regularly engage in and (b) whether physical efficacy acts as potential psychological mediator, mediating differences between groups of practitioners of different types of exercise and body image. To this end, a cross-sectional study design was chosen to compare ballroom dancers and practitioners of yoga against practitioners of team sports and individual sports. Non-exercisers served as reference group. As outlined in the *Introduction*, there is evidence from the literature that exercise and negative body image are related and that type of exercise and psychological factors might play a role and mediate this relationship. However, a comparison between different types of exercise including ballroom dance, yoga, team sports, or individual sports with regard to body image, negative body image in particular, is scarce. The cross-sectional study design of the present study will shed light on this by assessing negative body image in four groups reporting to engage in different types of exercise (including ballroom dance, yoga, team sports, or individual sports as the main exercise) and a non-exercising reference group. Assessment of body image included body dissatisfaction measures that relate to the perceptual, cognitive, and affective dimension of body image and that have been shown to be particularly indicative of negative body image.

Based on the available empirical evidence (outlined above), the following hypotheses were investigated:

1. The non-exercise group as reference group was hypothesized to show higher body dissatisfaction and higher body size distortion compared to the exercise groups.
2. Yoga practitioners and ballroom dancers (women and men) were expected to report a lower body dissatisfaction and body size distortion in comparison to the groups of team and individual sports practitioners.
3. Whether there is a difference between yoga practitioners and ballroom dancers in perceptual, cognitive, and affective measures related to negative body image is yet to be determined.
4. Practitioners of team sports were expected to differ in body dissatisfaction and body size distortion from practitioners of individual exercise.
5. Physical efficacy was expected to be a mediator that accounts for differences in type of exercise and body image measures.

In addition, body mass index (BMI), gender, and age were assessed as control variables. Moreover, body image is not independent from mental and physical health of practitioners. Therefore, it is important to determine to what degree and on which health- and exercise-related dimensions practitioners, belonging to the different exercise groups, differ. In order to consider these health-related variables, regular physical activity behavior, depressive symptoms, as well as mood state were assessed across the different exercise groups and the non-exercise group.

## Materials and Methods

### Participants

*N* = 365 participants registered in the online study. Participants were recruited via email and flyers at Ulm University and via the internet (Survey Circle and Facebook) to ensure broad study participation for all exercise and the non-exercise groups. In addition, yoga and (ballroom) dance schools as well as sports clubs in Germany were notified about the study. Recruitment of participants took place over the course of 2 months in the spring of 2017. Participation was voluntary and participants had to give written informed consent prior to study participation. Exclusion criterion was an age under 18. Inclusion criteria were an age equal or above 18 years and a proper understanding of the German language since the online questionnaire was set up in German. In total, 90 participants had to be excluded because of missing informed consent, unrealistic time to finish the survey questionnaire, age under 18, or a BMI under 16.5 (severe underweight) or higher than 40 (severe overweight). Another five participants were not considered because they did not fill out relevant questionnaires. Thus, 270 healthy adults (68 males, *M* = 29.06 years, *SD* = 12.94 years, range = 18–76) participated in the study.

### Design

The study design was a cross-sectional design. Selection of participants was based on standardized criteria including a set of questions to be filled out. Participants were asked about their type of exercise they engaged in. In addition, they were asked whether they exercised at least once a week. In case they stated to exercise less than once a week and not to be engaged in any type of exercise on a regular basis they were defined as non-exercisers. Participants who replied to engage in exercise were asked to indicate the type of exercise they carried out regularly. Moreover, as a manipulation check, they had to report all types of exercise they carried out besides their main exercise (i.e., the exercise they regularly engage in most often, in terms of exercise frequency and exercise duration). Criteria for the main exercise therefore were exercise being carried out most of the time, on most days of the week, and with the longest duration per session. Participants who reported to play for example soccer, football, or basketball in a team on a regular basis were assigned to the team sports exercise group. Individuals who replied to engage most often in exercises such as strength training or jogging (preferably on their own) were assigned to a separate exercise group labeled “individual exercise” group. This group was also compared to the non-exercise group. Group allegiance was therefore self-selected. Because of the study design, randomization of the participants to either of the groups was not possible but as described above, based on self-selection according to standardized self-report criteria. All measures included in this survey study were self-report measures presented in a survey online via the internet. The questions were provided via the online platform Unipark (Questback GmbH, 2016). The completion of the online questionnaire took approximately 40 min. Among all participants, 10 × 10€ coupons were raffled. The study was approved by the Local Ethics Committee of Ulm University^1^.

### Measures

#### Demographics

Informed consent was obtained and participants provided the necessary sociodemographic variables. All participants reported their height and weight to compute the BMI (*BMI* = mass/height^2^). The items about exercise allegiance were presented in the aforementioned way. Furthermore, participants were asked to state their expert level, the frequency and the duration of their main exercise.

#### Body Image: Perceptual, Cognitive, and Affective Dimensions

##### Perceptual body dissatisfaction, body size distortion, and self-classified weight

The *Body Image Assessment Scale* (BIAS-BD; Gardner et al., 2009) was used to assess the perceptual body image of a person. Participants are asked to rate their actual and desired body shape by choosing 1 out of 17 pictures that show either a male or female body. The bodies deviate from each other in size and shape, using 60% to 140% of the weight of anthropometric physical models of adult men and women. The deviance between actual and desired body shape ratings can be taken as an estimate of perceptual body dissatisfaction. The test–retest reliability for body dissatisfaction was *r_*tt*_* = 0.81 (Gardner et al., 2009). Concurrent validity is supported by the research of Gardner et al. (2009). In order to measure body size distortion, discrepancies between the reported BMI and the actual perceived shape, stated in the Body Image Assessment Scale (BIAS-BD; Gardner et al., 2009), were calculated. The concurrent validity of the scale between the actual reported BMI of the participants and perceived body image was *r* = 0.76 (Gardner et al., 2009). Of note, in the following analyses the deviation between actual and desired body shape (body dissatisfaction), as well as the deviation between actual reported BMI and perceived shape are given as absolute value. For this reason, the results should not be interpreted with regard to the direction of estimation. Self-classified weight was also assessed via standardized scales. The scale *Self-Classified Weight* of the *Multidimensional Body-Self Relations Questionnaire* (MBSRQ; Cash et al., 1986; German version by Vossbeck-Elsebusch et al., 2014) was used to examine the perception of one’s body weight from 1 = *very underweight* to 5 = *very overweight* (Cash, 2015).

##### Body dissatisfaction: cognitive and affective aspects

The cognitive and affective dimensions of body image assessing body dissatisfaction were assessed with three subscales of the *Multidimensional Body-Self Relations Questionnaire* (MBSRQ; Cash et al., 1986; German version by Vossbeck-Elsebusch et al., 2014) and the *Eating Disorder Inventory-2* (EDI-2; Garner, 1991; German version by Thiel et al., 1997). The Multidimensional Body-Self Relations Questionnaire (Cash et al., 1986) is a 69-item questionnaire assessing multiple cognitive, affective, and perceptual dimensions of body image, specifically concerning the overall evaluation of the body and appearance. One of the ten subscales is the Multidimensional Body-Self Relations Questionnaire—Appearance Scales (MBSRQ-AS), a 34-item scale, which was translated into German and validated by Vossbeck-Elsebusch et al. (2014). It is clustered into ten subscales, of which the following 3 were used in our study: *Appearance Evaluation* (e.g., “I am physically unattractive”; seven items), *Body Areas Satisfaction* (nine items), and *Self-Classified Weight* (two items, see above). The subscales measure appearance-related facets of body image. The items are rated on a five-point Likert-type scale. Appearance Evaluation ranges from 1 = *definitely disagree* to 5 = *definitely agree.* Higher scores indicate a higher affective body satisfaction, while lower scores reflect a higher body dissatisfaction. Body Areas Satisfaction measures affective body dissatisfaction/satisfaction and ranges from 1 = *very dissatisfied* to 5 = *very satisfied.* Higher scores depict a higher satisfaction with individual body parts. The internal consistency of the two scales as well as of the scale *Self Classified Weight* ranged from Cronbach’s alpha = 0.84 to Cronbach’s alpha = 0.90 (Vossbeck-Elsebusch et al., 2014). The range for the six-week test–retest reliability of the subscales was *r*_*tt*_ = 0.75 to *r*_*tt*_ = 0.80 (Vossbeck-Elsebusch et al., 2014). Convergent and discriminant validity is supported by the research of Cash (2015).

The Eating Disorder Inventory-2 (Garner, 1991; translated to German and validated by Thiel et al., 1997) is a widely used measure to assess different cognitive and behavioral dimensions of EDs. The inventory consists of eleven scales with ratings on a six-point Likert-type scale (1 = *never* to 6 = *always*). In the present study, the subscale *Body Dissatisfaction* (e.g., “I think that my stomach is too big”; eight items), measuring the cognitive dimension of body dissatisfaction, was used. The internal consistency was Cronbach’s alpha = 0.88 in a subsample of anorectic and bulimic patients. Test–retest reliability was *r_*tt*_* = 0.89 (Thiel and Paul, 2006).

#### Physical Efficacy

The subscale *Physical efficacy* (SKEF; e.g., “I am strong” [translation by the author]; ten items) of the *Frankfurt Body Concept Scales* (Frankfurter Körperkonzeptskalen; FKKS; German version; Deusinger, 1998) was used to measure the perception of one’s physical efficacy. The FKKS questionnaire (Deusinger, 1998) assesses cognitive, affective, and behavioral attitudes toward the body. This 64-item scale measures nine different body concepts via nine different subscales. The questionnaire only exists in the German language. The *Physical efficacy* subscale (SKEF) measures perceived strength and agility of the body. Participants are asked to rate the items on a six-point Likert-type scale ranging from 1 = *completely agree* [translation by the author] to 6 = *completely disagree* [translation by the author]. High scorers tend to have a higher physical efficacy. The internal consistency of the subscale in our study was Cronbach’s alpha = 0.87. Convergent validity was found in a study by Pöhlmann et al. (2014) where subscales of the FKKS were highly correlated with subscales of the *Dresdner Body Image Questionnaire (Dresdner Körperbildfragebogen [translation by the author]*, DKB-35; Thiel, 2007), all of them covering comparable dimensions.

##### Regular physical activity behavior, mood, and depression

Regular physical activity was assessed via the *Global Physical Activity Questionnaire* (GPAQ; World Health Organization [WHO], 2013). This 19-item scale measures the frequency and duration (in hours and minutes) of physical activity at work, travel to and from places and during leisure time, but also sedentary time. Mood was assessed via visual analog scales, where participants rated state as actual mood and trait as mood during the last two weeks with smileys. In addition, they filled in the *Positive and Negative Affect Scale* (PANAS; Watson et al., 1988; German version by Krohne et al., 1996) to assess habitual (trait) positive and negative affect. The *Patient Health Questionnaire-2* (PHQ-2; Kroenke et al., 2003) was used to screen depressive symptomatology, according to the diagnostic criteria of the Diagnostic and Statistical Manual of Mental Disorders (DSM-IV; Saß et al., 1996). A cut-off score of 3 and above in the PHQ-2 has been shown to be indicative of depressive disorder.^2^

### Data Analysis and Statistics

All statistical tests and analyses were conducted with RStudio (Version 1.2.5033, R Studio Team, 2019). Differences between the different groups (exercise groups, reference group of non-exercisers) on the variables age, BMI, expertise, exercise frequency, and exercise duration were statistically tested with a one-way analysis of variance (ANOVA). Group differences in body image and in mental-health related variables including depression, mood, and physical activity were tested with multiple linear regression analysis. In order to compare groups based on our hypotheses, orthogonal contrasts of the between-subject factor *group* (yoga, ballroom dance, team sports, individual sports, and non-exercisers) were chosen. In line with the hypotheses (see section “INTRODUCTION”), the individual contrasts compared the effects between the non-exercise group versus all exercise groups (contrast 1), between the yoga group and dance group versus team sports and individual sports groups (contrast 2), between the yoga group versus ballroom dance group (contrast 3), and between the team versus individual sports groups (contrast 4). Of note, following the logic of orthogonal contrasts, the four contrasts allow for the comparison of hypothesis 1 that non-exercisers show a higher body dissatisfaction and body size distortion compared to all exercise groups (contrast 1); hypothesis 2 that yoga and ballroom dancers show a lower body dissatisfaction and body size distortion compared to team and individual exercise practitioners (contrast 2), also whether there is a difference between yoga practitioners and ballroom dancers (hypothesis 3, contrast 3) and hypothesis 4 that there is a difference in body image between team and individual exercise practitioners (contrast 4).

The variables sex and age were added into the model to control for significant differences between groups on these variables. The assumptions of multiple linear regression were tested with the possibility of referring to a bootstrapping procedure according to Field et al. (2012, pp. 298–301). Deviations from normality of the residuals were ignored following the central limit theorem because all groups consisted of more than 30 participants.

**FIGURE 1:**
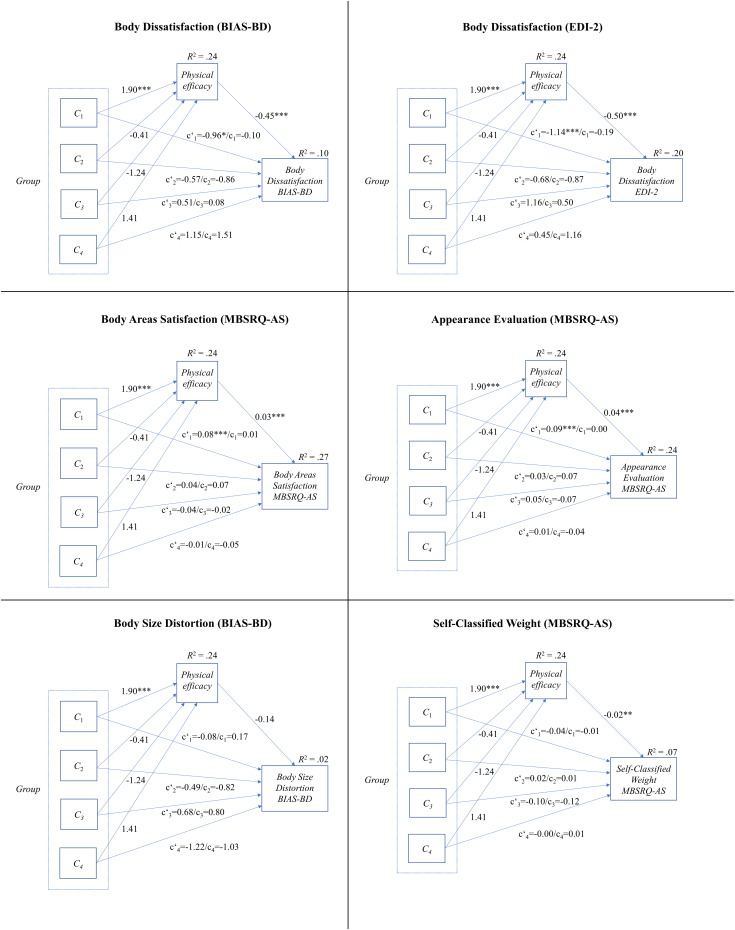
Mediation models of group contrasts, physical efficacy, and perceptual, cognitive, and affective body image measured via the *Body Image Assessment Scale-Body Dimensions* (BIAS-BD; Gardner et al., 2009), the *Eating Disorder Inventory-2* (EDI-2, Garner, 1991; German version by Thiel et al., 1997), and the *Multidimensional Body-Self Relations Questionnaire-Appearance Scales* (MBSRQ-AS; Cash et al., 1986; German version by Vossbeck-Elsebusch et al., 2014). Contrast 1: exercise versus non-exercise; contrast 2: yoga and ballroom dance versus team and individual sports; contrast 3: yoga versus ballroom dance; contrast 4: team versus individual sports. ^∗^*p* ≤ 0.05, ^∗∗^*p* < 0.01, ^∗∗∗^*p* < 0.001.

Mediation analyses with physical efficacy as psychological factor mediating the relationship between (a) possible differences between exercise groups (contrasts 2–4) and (b) differences between exercise groups and the non-exercise group (contrast 1) with respect to the body image variables body dissatisfaction, body size distortion, and self-classified weight were carried out (also see Figure 1). The steps for mediation analysis were performed according to the guidelines by Zhao et al. (2010). Following Zhao et al. (2010), only a bootstrap test of the indirect effect *a* × *b* is necessary in order to test mediation. This should replace individual testing of path *a* (regressing mediator on independent/exogenous variable), path *b* (mediator to affect dependent variable), and path *c’* (independent/exogenous variable affecting dependent variable) as well as replace the use of the Sobel test for the indirect path *a* × *b*. As recommended, we focused on the significance of the indirect effect in order to find a mediation effect and on the direct effect (Zhao et al., 2010; Rucker et al., 2011). The PROCESS makro by Hayes (2018) with bootstrapping was used in order to test the indirect effects in the mediation analysis with independent variable *group* containing five levels and the contrasts. 95% confidence intervals are reported. The *p*-value was set to *p* ≤ 0.05. For linear regression, multiple comparisons were controlled for via the false discovery rate (FDR) by Benjamini and Hochberg (1995).

## Results

### Sample Characteristics

#### Sample Size, Age, Gender, BMI, and Education

The five groups comprising the different exercise groups and the non-exercise group were almost equal in size (yoga: *n* = 44; dance: *n* = 40; team sports: *n* = 56; individual sports: *n* = 65; non-exercise: *n* = 65). The distribution of sex (male versus female) differed between groups (yoga: *n* = 5 males [11.36%], ballroom dance: *n* = 18 males [45.00%], team sports: *n* = 15 males [26.79%], individual sports: *n* = 18 males [27.69%], non-exercise: *n* = 12 males [18.46]). Mean age over all groups was 29.06 (*SD* = 12.94). Age ranged from 18 to 76 years and varied across groups, *F*(4,265) = 3.54, *p* < 0.01, ω^2^ = 0.19. Age was significantly different between the yoga and the non-exercise group, *p* < 0.01, *d* = 0.77, i.e., the participants of the yoga group were on average older than the participants of the ballroom dance group. All groups were comparable in self-reported BMI, *F*(4,123.82) = 1.32, *p* = 0.265, as shown in Table 1. Self-reported BMI ranged from 16.76 to 37.20 (*M* = 22.89, *SD* = 3.76). Most participants were highly educated (completed professional education: *n* = 39, Abitur: *n* = 129, bachelor/master degree: *n* = 69, doctorate: *n* = 6, chair: *n* = 1). Only five participants stated to have no degree (yet).

**TABLE 1:** Participant characteristics: Mean scores and standard deviations of demographic variables in dependence of group.

	**Group**				
	**Yoga**	**Ballroom dance**	**Team sports**	**Individual sports**	**Non-exercise**
**Age**	34.20 (13.14)	26.95 (13.59)	27.93 (12.10)	30.97 (15.33)	25.94 (8.89)
**BMI**					
Male	23.65 (2.67)	24.46 (4.33)	23.78 (2.21)	24.85 (4.65)	23.83 (3.67)
Female	21.82 (3.14)	22.54 (4.40)	22.34 (3.00)	21.81 (2.81)	23.48 (4.57)
Total	22.03 (3.12)	23.40 (4.42)	22.73 (2.87)	22.65 (3.64)	23.54 (4.39)
**Exercise**					
GPAQ Total physical activity per day (in minutes)	178.62 (251.03)	131.52 (151.35)	145.82 (341.11)	153.12 (330.21)	106.46 (317.26)
Expertise	57.02 (73.78)	78.10 (80.74)	91.55 (101.84)	99.99 (130.04)	-
Frequency	3.16 (0.94)	2.88 (0.82)	3.25 (0.82)	3.22 (0.82)	-
Duration	70.46 (31.93)	114.75 (50.95)	92.32 (30.05)	67.92 (31.01)	-
**Sedentary time**					
GPAQ Sedentary time per day (in minutes)	396.05 (210.29)	398.54 (248.84)	379.02 (181.44)	423.53 (243.18)	537.72 (265.91)

*BMI = body mass index. Expertise in months; frequency in times per week; duration in minutes per session. Global Physical Activity Questionnaire (GPAQ; World Health Organization [WHO], 2013).*

#### Regular Physical Activity

Mean physical activity per day comprising work activity, leisure time activity, and activity while traveling from and to places over all groups (exercise groups and reference groups) was 2.35 h per day, amounting to 16.48 h per week. The non-exercise group showed the lowest regular physical activity per day in the Global Physical Activity Questionnaire (World Health Organization [WHO], 2013). Highest regular physical activity per day was found in the yoga group. There were no significant differences between groups, *F*(4,265) = 0.42, *p* = 0.797. Sedentary time for all groups was 7.23 h per day. The non-exercise group showed the highest sedentary time per day compared to all exercise groups (contrast 1), *B* = −28.16, *SE*_*B*_ = 6.61, *t*(263) = −4.26, *p* < 0.001. All other contrasts were not significant [yoga and dance versus team and individual sports: *B* = −0.39, *SE*_*B*_ = 16.28, *t*(263) = −0.02, *p* = 0.981; yoga versus dance: *B* = 10.58, *SE*_*B*_ = 26.17, *t*(263) = 0.40, *p* = 0.801; team sports versus individual sports: *B* = −28.00, *SE*_*B*_ = 20.91, *t*(263) = −1.34, *p* = 0.255]. The amount of variance explained by the variables in sedentary time was significant, *F*(6,263) = 4.18, *p* < 0.001, *R_*adj*_^2^* = 0.07, *f^2^* = 0.10.

#### Exercise Frequency and Exercise Duration Across Exercise Groups

As shown in Table 1, the four exercise groups participated in their main exercise on average 3.15 (*SD* = 0.85) times per week. Frequency did not vary between groups, *F*(3,201) = 1.80, *p* = 0.148. Participants carried out their main exercise with an average duration of 48.09 (*SD* = 73.10) min per week. The average duration differed between exercise groups, *F*(3,99.97) = 3.75, *p* < 0.001. Differences were found between the yoga group and the ballroom dance group, *p* < 0.001, *d* = −1.05, and between the yoga group and the team sports group, *p* < 0.01, *d* = −0.71. The yoga practitioners had a shorter duration per session. Furthermore, the ballroom dance group reported a longer duration of each individual dancing session compared to the team sports group, *p* < 0.05, *d* = 0.56, and the individual sports group, *p* < 0.001, *d* = 1.18. Individual sports sessions were also shorter in duration compared to team sports sessions, *p* < 0.001, *d* = 0.80. Participants of the exercise groups replied to engage in their main exercise already on average for the last 48.03 (*SD* = 110.89) months. There was no significant difference between the exercise groups with regard to expertise, *F*(3,201) = 1.67, *p* = 0.176.

### Body Image: Perceptual, Cognitive, and Affective Factors

Descriptive statistics of the different body image dimensions are summarized in Table 2. Overall, the non-exercise group showed the highest body dissatisfaction on all self-report body image dimensions (perceptual, cognitive, and affective). The ballroom dance group had the most favorable scores on the Body Image Assessment Scale (BIAS-BD; Gardner et al., 2009), measuring dissatisfaction with body size, weight and shape, the Eating Disorder Inventory-2 (EDI-2) subscale, measuring body dissatisfaction (Garner, 1991; German version by Thiel et al., 1997), and the Body Areas Satisfaction scale, measuring satisfaction with discrete aspects (body parts, body areas) of one’s appearance (MBSRQ-AS; Cash et al., 1986; German version by Vossbeck-Elsebusch et al., 2014). Appearance evaluation (Appearance Evaluation; MBSRQ-AS; Cash et al., 1986; German version by Vossbeck-Elsebusch et al., 2014) as affective dimension of body image, measuring feelings of physical attractiveness, was highest for the yoga group. Self-classified weight was highest in the non-exercise group in the MBSRQ-AS (Self-Classified Weight; Cash et al., 1986; German version by Vossbeck-Elsebusch et al., 2014). The individual exercise group showed the highest body size distortion rated via the Body Image Assessment Scale (BIAS-BD; Gardner et al., 2009). Lowest body size distortion was found for the ballroom dance group.

**TABLE 2:** Descriptive statistics: Mean scores, standard deviations (SDs in brackets) and group size (*n*) of body image questionnaires in dependence of group.

	**Questionnaire (range)**	**Group**					
		**Yoga**	**Ballroom dance**	**Team sports**	**Individual sports**	**Non-exercise**	**Total**
**Perceptual Body Image**	**BIAS-BD Body Dissatisfaction (0–40)**	12.49 (10.43) (*n* = 44)	11.46 (11.24) (*n* = 40)	14.25 (10.91) (*n* = 55)	11.95 (12.27) (*n* = 65)	17.31 (14.72) (*n* = 64)	13.72 (12.33) (*n* = 268)
	**BIAS-BD Body Size Distortion (0–40)**	14.13 (12.56) (*n* = 44)	12.78 (8.54) (*n* = 40)	13.22 (10.42) (*n* = 55)	15.66 (12.61) (*n* = 65)	14.34 (11.16) (*n* = 65)	14.16 (11.25) (*n* = 269)
	**MBSRQ-AS Self-Classified Weight (1–5)**	3.02 (0.43) (*n* = 44)	3.22 (0.60) (*n* = 38)	3.08 (0.39) (*n* = 54)	3.09 (0.60) (*n* = 64)	3.29 (0.74) (*n* = 64)	3.14 (0.58) (*n* = 264)
**Cognitive Body Image**	**EDI-2 Body Dissatisfaction (9–54)**	26.23 (8.69) (*n* = 44)	23.91 (10.93) (*n* = 35)	26.89 (10.22) (*n* = 54)	25.98 (9.20) (*n* = 63)	31.44 (10.49) (*n* = 62)	27.24 (10.14) (*n* = 258)
**Affective Body Image**	**MBSRQ-AS Body Areas Satisfaction (1–5)**	3.62 (0.50) (*n* = 44)	3.69 (0.57) (*n* = 39)	3.57 (0.58) (*n* = 54)	3.59 (0.51) (*n* = 64)	3.24 (0.66) (*n* = 64)	3.52 (0.59) (*n* = 265)
	**MBSRQ-AS Appearance Evaluation (1–5)**	3.73 (0.61) (*n* = 44)	3.63 (0.79) (*n* = 40)	3.63 (0.69) (*n* = 55)	3.61 (0.73) (*n* = 65)	3.21 (0.90) (*n* = 64)	3.54 (0.78) (*n* = 268)

*Body image was assessed via the Body Image Assessment Scale-Body Dimensions (BIAS-BD, absolute value of deviation in percent; Gardner et al., 2009) for body dissatisfaction and body size distortion, the Eating Disorder Inventory-2 (EDI-2, sum score; Garner, 1991; German version by Thiel et al., 1997) for body dissatisfaction and the Multidimensional Body-Self Relations Questionnaire-Appearance Scales (MBSRQ-AS, mean score; Cash et al., 1986; German version by Vossbeck-Elsebusch et al., 2014) for body dissatisfaction/body satisfaction and self-classified weight.*

#### Perceptual Body Dissatisfaction, Body Size Distortion, and Self-Classified Weight

Multiple regression analysis for perceptual body dissatisfaction, measured via the Body Image Assessment Scale (Gardner et al., 2009), revealed a significant effect of the contrast between the non-exercise group and all exercise groups, *B* = −1.00, *SE*_*B*_ = 0.36, *t*(261) = −2.80, *p* < 0.01. The non-exercise group showed a higher body dissatisfaction (Gardner et al., 2009). All other contrasts were not significant [yoga and dance versus team and individual sports: *B* = −0.60, *SE*_*B*_ = 0.87, *t*(261) = −0.68, *p* = 0.346, yoga versus dance: *B* = 0.18, *SE*_*B*_ = 1.40, *t*(261) = 0.13, *p* = 0.900, team versus individual sports: *B* = 1.24, *SE*_*B*_ = 1.13, *t*(261) = 1.10, *p* = 0.249]. The amount of variance explained by the variables in perceptual body dissatisfaction was not significant, *F*(6,261) = 1.65, *p* = 0.132, *R_*adj*_^2^* = 0.02, *f^2^* = 0.04.

There was no significant difference between groups with regard to body size distortion of the Body Image Assessment Scale [BIAS-BD; Gardner et al., 2009; exercise groups versus non-exercise: *B* = −0.11, *SE*_*B*_ = 0.33, *t*(262) = −0.35, *p* = 0.425; yoga and dance versus team and individual sports: *B* = −0.53, *SE*_*B*_ = 0.81, *t*(262) = −0.66, *p* = 0.359; yoga versus dance: *B* = 0.16, *SE*_*B*_ = 1.29, *t*(262) = 0.12, *p* = 0.0.904; team versus individual sports: *B* = −1.11, *SE*_*B*_ = 1.04, *t*(262) = −1.07, *p* = 0.331]. All in all, the variables did not explain a significant amount of variance in perceptual body size distortion, measured via the BIAS-BD (Gardner et al., 2009), *F*(6,262) = 0.71, *p* = 0.638, *R_*adj*_^2^* = −0.01, *f^2^* = 0.02.

Significant differences were found for self-classified weight (Self-Classified Weight; MBSRQ-AS; Cash et al., 1986; German version by Vossbeck-Elsebusch et al., 2014). These differences were detected between the non-exercise and the exercise groups, *B* = −0.04, *SE*_*B*_ = 0.02, *t*(257) = −2.51, *p* < 0.05. The non-exercise group showed a higher perceived body weight than the exercise groups taken together. On top of that, the yoga group had a lower self-classified weight in contrast to the ballroom dance group, *B* = −0.16, *SE*_*B*_ = 0.07, *t*(257) = −2.44, *p* < 0.05. The variables included explained a significant amount of variance in self-classified weight, *F*(6,257) = 3.26, *p* < 0.01, *R_*adj*_^2^* = 0.05, *f^2^* = 0.08. No significant effect was found for the other contrasts and factors [yoga and dance versus team and individual sports: *B* = 0.02, *SE*_*B*_ = 0.04, *t*(257) = 0.39, *p* = 0.817; team versus individual sports: *B* = 0.01, *SE*_*B*_ = 0.05, *t*(257) = 0.23, *p* = 0.818].

#### Cognitive and Affective Body Dissatisfaction

A similar pattern as in perceptual body dissatisfaction was found for cognitive body dissatisfaction as the key factor of a possible risk of ED in the EDI-2 (Garner, 1991; German version by Thiel et al., 1997) with a significant difference between the non-exercise group and all four exercise groups taken together, *B* = −1.04, *SE*_*B*_ = 0.29, *t*(251) = −3.65, *p* < 0.001. The non-exercise group showed a higher cognitive body dissatisfaction compared to the exercise groups. The variables explained a significant amount of variance, *F*(6,251) = 6.01, *p* < 0.001, *R_*adj*_^2^* = 0.11, *f^2^* = 0.14. All other contrast effects were not significant [yoga and dance versus team and individual sports: *B* = −0.62, *SE*_*B*_ = 0.70, *t*(251) = −0.88, *p* = 0.333; yoga versus dance: *B* = −0.05, *SE*_*B*_ = 1.14, *t*(251) = −0.05, *p* = 0.963; team versus individual sports: *B* = 0.51, *SE*_*B*_ = 0.89, *t*(251) = 0.57, *p* = 0.396].

There were also significant differences between the different groups in affective body areas satisfaction (Body Areas Satisfaction; MBSRQ-AS; Cash et al., 1986; German version by Vossbeck-Elsebusch et al., 2014), *F*(6,258) = 3.78, *p* < 0.01, *R_*adj*_^2^* = 0.06, *f^2^* = 0.09. The main effect for the contrast between the non-exercise group and the exercise groups was significant, *B* = 0.08, *SE*_*B*_ = 0.02, *t*(258) = 4.54, *p* < 0.001. The non-exercise group had lower body satisfaction/higher body dissatisfaction scores for this affective component of body image than the exercise group overall. All other contrasts did not show a significant effect on affective body areas satisfaction [yoga and dance versus team and individual sports: *B* = 0.04, *SE*_*B*_ = 0.04, *t*(258) = 0.94, *p* = 0.351; yoga versus dance: *B* = −0.02, *SE*_*B*_ = 0.07, *t*(258) = −0.30, *p* = 0.831; team versus individual sports: *B* = −0.01, *SE*_*B*_ = 0.05, *t*(258) = −0.21, *p* = 0.416].

The effect on appearance evaluation as affective dimension (Appearance Evaluation; MBSRQ-AS; Cash et al., 1986; Vossbeck-Elsebusch et al., 2014) was significant for the contrast between the non-exercise group and all other groups, *B* = 0.09, *SE*_*B*_ = 0.02, *t*(261) = 4.14, *p* < 0.001. The non-exercise group showed lower body satisfaction/higher body dissatisfaction scores. All other effects were not significant [yoga and dance versus team and individual sports: *B* = 0.03, *SE*_*B*_ = 0.05, *t*(261) = 0.60, *p* = 0.322; yoga versus dance: *B* = 0.11, *SE*_*B*_ = 0.09, *t*(261) = 1.27, *p* = 0.289; team versus individual sports: *B* = 0.00, *SE*_*B*_ = 0.07, *t*(261) = -0.01, *p* = 0.496]. The variables included explained a significant amount of variance in affective body satisfaction/dissatisfaction, *F*(6,261) = 3.82, *p* < 0.01, *R_*adj*_^2^* = 0.06, *f^2^* = 0.09.

### Mediation Analysis

Mediation analyses were performed in order to analyze whether the results found for the different dimensions of body image (perceptual body dissatisfaction, body size distortion, self-classified weight, and cognitive and affective body dissatisfaction) concerning differences between groups are mediated by physical efficacy. As can be seen in Table 3, a significant indirect effect emerged for the first contrast (exercise versus non-exercise) for perceptual, cognitive, and affective dimensions of body dissatisfaction. The direct effects for these dimensions (contrast 1) were not significant (see Figure 1). According to Zhao et al. (2010), this implies an indirect-only mediation. No mediation was detected for contrasts 2 (yoga and ballroom dance versus team and individual sports), 3 (yoga versus ballroom dance), and 4 (team versus individual sports). The indirect effect was significant for the body image variable self-classified weight as measured with the MBSRQ-AS (Cash et al., 1986; German version by Vossbeck-Elsebusch et al., 2014). The indirect effect concerning body size distortion in the Body Image Assessment Scale (Gardner et al., 2009) was not significant, hence, no mediation effect could be detected. Figure 1 depicts the size of effects for the different paths.

**TABLE 3:** Indirect and direct effects of mediation analysis for the factors contrast as exogenous variable (contrast 1: exercise versus non-exercise; contrast 2: yoga and ballroom dance versus team and individual sports; contrast 3: yoga versus ballroom dance; contrast 4: team versus individual sports), body image variables and physical efficacy as mediator.

	**Effect**	**Perceptual Body Image**			**Cognitive Body Image**	**Affective Body Image**	
		**BIAS-BD Body Dissatisfaction**	**BIAS-BD Body Size Distortion**	**MBSRQ-AS Self-Classified Weight**	**EDI-2 Body Dissatisfaction**	**MBSRQ-AS Body Areas Satisfaction**	**MBSRQ-AS Appearance Evaluation**
**Contrast 1**	**Indirect effect** a*b [95% CI]	**−0.85 [−1.41, −0.29]**	**−**0.27 [-0.81, 0.29]	**−0.03 [−0.05, −0.00]**	**−0.96 [−1.43, −0.47]**	**0.06 [0.03, 0.09]**	**0.08 [0.04, 0.12]**
	**Direct effect** *c* (*p* value)	**−**0.10 (*p* = 0.955)	0.17 (*p* = 0.650)	**−**0.01 (*p* = 0.833)	**−**0.19 (*p* = 0.629)	0.01 (*p* = 0.670)	0.01 (*p* = 0.826)
**Contrast 2**	**Indirect effect** a*b [95% CI]	0.18 [**−**0.47, 0.80]	0.06 [**−**0.23, 0.33]	0.01 [**−**0.02, 0.03]	0.20 [**−**0.48, 0.89]	**−**0.01 [**−**0.06, 0.03]	**−**0.02 [**−**0.08, 0.04]
	**Direct effect** *c* (*p* value)	**−**0.86 (*p* = 0.475)	**−**0.82 (*p* = *0.496*)	0.01 (*p* = 0.833)	**−**0.87 (*p* = *0.293*)	0.07 (*p* = 0.126)	0.07 (*p* = 0.321)
**Contrast 3**	**Indirect effect** a*b [95% CI]	0.55 [**−**0.46, 1.57]	0.18 [**−**0.38, 0.71]	0.02 [**−**0.02, 0.06]	0.62 [**−**0.51, 1.75]	**−**0.04 [**−**0.12, 0.03]	**−**0.05 [**−**0.15, 0.05]
	**Direct effect** *c* (*p* value)	0.08 (*p* = 0.955)	0.80 (*p* = 0.632)	**−**0.12 (*p* = 0.109)	0.50 (*p* = 0.629)	**−**0.02 (*p* = 0.755)	0.07 (*p* = 0.576)
**Contrast 4**	**Indirect effect** a*b [95% CI]	**−**0.63 [**−**1.42, 0.19]	**−**0.20 [**−**0.67, 0.27]	**−**0.02 [**−**0.05, 0.01]	**−**0.71 [**−**1.53, 0.11]	0.05 [**−**0.01, 0.10]	0.06 [**−**0.01, 0.13]
	**Direct effect** *c* (*p* value)	1.51 (*p* = 0.345)	**−**1.03 (*p* = 0.496)	0.01 (*p* = 0.833)	1.16 (*p* = 0.293)	**−**0.05 (*p* = 0.445)	**−**0.04 (*p* = 0.650)

*Significant results are printed in bold. Body image was assessed via the Body Image Assessment Scale-Body Dimensions (BIAS-BD; Gardner et al., 2009) for body dissatisfaction and body size distortion, the Eating Disorder Inventory-2 (EDI-2; Garner, 1991; German version by Thiel et al., 1997) for body dissatisfaction, and the Multidimensional Body-Self Relations Questionnaire-Appearance Scales (MBSRQ-AS; Cash et al., 1986; German version by Vossbeck-Elsebusch et al., 2014) for body dissatisfaction, body satisfaction, and self-classified weight.*

### Mental Health: Mood and Depression Risk

As can be seen in Table 4, the non-exercise group showed the highest score in the PHQ-2 (Kroenke et al., 2003) which screened for depressive symptoms. The mean score of the non-exercise group was significantly higher compared to the mean score averaged across all exercise groups (contrast 1), *B* = −0.10, *SE*_*B*_ = 0.04, *t*(251) = −2.93, *p* < 0.05. The ballroom dance group and the individual sports group showed the lowest scores on the PHQ-2. There was no significant difference for the other contrasts [yoga and dance versus team and individual sports: *B* = 0.00, *SE*_*B*_ = 0.09, *t*(251) = 0.01, *p* = 0.990; yoga versus dance: *B* = 0.15, *SE*_*B*_ = 0.14, *t*(251) = 1.06, *p* = 0.549; team sports versus individual sports: *B* = 0.11, *SE*_*B*_ = 0.11, *t*(251) = 1.01, *p* = 0.549]. The amount of variance explained by the variables in depression was not significant, *F*(6,251) = 2.03, *p* = 0.063, *R_*adj*_^2^* = 0.02, *f^2^* = 0.05. It has to be stated that all mean depression scores for the groups were in the lowest third of range indicating low depression in this sample. Concerning positive affect, ballroom dancers showed the highest positive affect. The non-exercise group reported the lowest positive affect which was significantly lower compared to the exercise groups, *B* = 0.08, *SE*_*B*_ = 0.02, *t*(250) = 4.46, *p* < 0.001. There was no significant effect of any of the other contrasts [yoga and dance versus team and individual sports: *B* = 0.06, *SE*_*B*_ = 0.05, *t*(250) = 1.26, *p* = 0.294; yoga versus dance: *B* = −0.10, *SE*_*B*_ = 0.08, *t*(250) = −1.26, *p* = 0.294; team sports versus individual sports: *B* = 0.06, *SE*_*B*_ = 0.06, *t*(250) = 1.09, *p* = 0.324]. The amount of variance explained by the variables in positive trait affect was significant, *F*(6,250) = 4.47, *p* < 0.001, *R_*adj*_^2^* = 0.08, *f^2^* = 0.11. The yoga group showed the highest negative trait affect, while the team sports group had the lowest negative trait affect. None of the effects of the contrasts were significant [exercise groups versus non-exercise group: *B* = −0.03, *SE*_*B*_ = 0.02, *t*(250) = −1.21, *p* = 0.400; yoga and dance versus team and individual sports: *B* = 0.12, *SE*_*B*_ = 0.06, *t*(250) = 2.07, *p* = 0.139; yoga versus dance: *B* = 0.12, *SE*_*B*_ = 0.09, *t*(250) = 1.34, *p* = 0.400; team sports versus individual sports: *B* = −0.04, *SE*_*B*_ = 0.07, *t*(250) = −0.55, *p* = 0.794]. The amount of variance explained by the variables in negative trait affect was not significant, *F*(6,250) = 1.57, *p* = 0.156, *R_*adj*_^2^* = 0.01, *f^2^* = 0.04.

**TABLE 4:** Mean scores and standard deviations (SDs in brackets) of mental health variables in dependence of group.

**Questionnaire (range)**	**Group**					
	**Yoga**	**Ballroom dance**	**Team sports**	**Individual sports**	**Non-exercise**	**Total**
**PHQ-2 Depression (0–6)**	1.43 (1.09)	1.14 (1.14)	1.41 (1.06)	1.18 (1.01)	1.82 (1.50)	1.42 (1.20)
**PANAS Trait Positive affect (1–5)**	3.42 (0.66)	3.59 (0.54)	3.44 (0.67)	3.32 (0.64)	3.00 (0.64)	3.32 (0.66)
**PANAS Trait Negative affect (1–5)**	2.18 (0.79)	1.92 (0.81)	1.77 (0.67)	1.86 (0.77)	2.07 (0.76)	1.95 (0.77)

*The following questionnaires were used as control variables: Patient Health Questionnaire-2 (PHQ-2; Kroenke et al., 2003) and Positive and Negative Affect Schedule (PANAS; Watson et al., 1988; German version by Krohne et al., 1996).*

## Discussion

In the current study, self-report measures were used to assess perceptual, cognitive, and affective aspects of a negative body image and potential differences therein between different exercise groups, including practitioners of ballroom dance, yoga, team or individual sports, and a non-exercise group. The focus was on measures of negative body image to shed further light on possible differential effects of regular exercise on perceptual, cognitive, and affective body image dimensions, specifically body dissatisfaction and body size distortion. Furthermore, self-reported regular physical activity, mood, and depressive symptoms were explored, and physical efficacy was tested as one possible mediating variable for differences in body image between the exercise and non-exercise groups.

Of note, the design of this study was cross-sectional and participants filled in questionnaires in an online survey. Therefore, selection of the participants to the different exercise groups was based solely on self-report. Only the main exercise reported by the participants as the most executed one was considered to form the exercise groups. Carrying out other exercises in addition to the main exercise is quite common in the general population. Therefore, one advantage of the current selection procedure was that the present study in comparison to many previous cross-sectional exercise studies reported and controlled for this by asking the participants in detail about all exercises they practiced and then building groups on the main exercise with highest frequency. This heightens the generalizability and ecological validity of the findings when describing individual differences between exercisers and non-exercisers.

### Types of Exercise and Body Image

Underlining previous results in the body image literature, people reporting no particular regular type of exercise and reporting to exercise less than once a week (i.e., participants of the non-exercise group) showed a higher negative body image in comparison to people reporting to carry out a certain type of exercise regularly (more than once a week). This difference between engaging in a particular type of exercise versus no exercise engagement was found for perceptual, cognitive, as well as affective measures measuring body dissatisfaction. This association between all facets of a negative body image and no regular exercise complies with results reported in a recent review (Sabiston et al., 2019), which found this pattern in the majority of studies included in the review (> 80%). Thus, in line with this recent review, the present results support the importance of carrying out exercise at least once a week in order to lower body dissatisfaction. Moreover, the current results underline that basically all dimensions (perceptual, cognitive, and/or affective) of body image differ between non-exercisers and individuals who carry out regular exercise including different types of exercise such as yoga, ballroom dance, team sports, or individual sports. This main difference between groups was found independent of sex and age, since sex and age were included as covariates. This is important because sex and age are known to highly influence body image (Demarest and Allen, 2000). Both sex and age differed between groups in the current study. In case of yoga, there was a gender bias, with more women than men in the yoga group. No gender bias occurred in the group of ballroom dancers, possibly because ballroom dance is a type of exercise carried out with a partner, which explains why the amount of men in this group was higher compared to other exercise groups such as yoga.

There was no significant difference between exercise groups and the non-exercise group with regard to perceptual body size distortion in the Body Image Assessment Scale (Gardner et al., 2009). This might be explainable by the assessment of body size distortion with the traditional figure rating scale procedure using the Stunkard scales. The figure rating procedure uses BMI adjusted silhouettes to measure discrepancies between actual and ideal body image that does not take body musculature in consideration. Body musculature, however, might be a relevant body feature for individuals engaged in regular exercise.

All groups had a normal self-classified weight (Self-Classified Weight; MBSRQ-AS; Cash et al. (1986); German version by Vossbeck-Elsebusch et al., 2014) corresponding to an actual normal weight according to their reported BMI. Interestingly, non-exercisers perceived themselves as having a higher weight compared to exercise practitioners. On top of that, a higher self-classified weight for ballroom dancers compared to yoga practitioners was found. This cannot be due to a gender bias, since sex was included as control variable. Non-exercisers indeed had the highest actual reported BMI followed by ballroom dancers while yoga practitioners had the lowest self-classified weight of all groups. This shows that even little differences in actual BMI, although not significant, might be perceivable, and with regard to self-classified weight, non-exercisers are as good as exercise groups in the perception of body weight.

There was no advantage of yoga and ballroom dance over team and individual exercise with regard to self-report measures of body dissatisfaction and body size distortion. Positive effects of yoga on body image were reported in numerous studies (Mahlo and Tiggemann, 2016; e.g., Neumark-Sztainer et al., 2018). On top of that, in a survey study investigating college students, body image differed as a function of preferences for team versus individual exercise (e.g., Morano et al., 2011). In the current study, there were no significant differences between team and individual sports exercisers. The participants of the group of individual sports had different exercise preferences. Accordingly, it could be that someone working out in a gym might have completely different body image-related experiences than someone going outside for a run. Likewise, people working out in groups in a fitness setting and people working out completely on their own might differ in their body image. As mentioned, the individuals included in the individual sports group were engaged in rather heterogeneous exercises. Nevertheless, treating the individual exercise group as a separate type of exercise group in our group comparisons makes sense because according to their reported exercise preferences, these participants neither mentioned to engage in yoga, ballroom dance, or team sports, nor were they non-exercisers. The current group comparisons suggest that, at least with respect to a negative body image, team sports or individual sports do not impact body image differently. To further explore the relationship between body image and different types of exercise within the individual sports group, future studies are needed.

The descriptive results of the current study depicting healthy adults of average age (mean age: 29.06) indicate a lower body dissatisfaction and perceptual body size distortion for ballroom dancers compared to all other groups, although group differences were not significant. Concerning ballroom dance, only a handful of studies exist up to date, and these mainly investigated elderly or physically impaired people (e.g., Gomes da Silva Borges et al., 2012; Kunkel et al., 2017).

So far, there is not enough evidence from previous studies regarding body image and ballroom dance. The present study compared ballroom dance to other types of exercise such as yoga. Yoga and ballroom dance differ with regard to several exercise aspects. These aspects are of relevance when investigating the effects of different types of exercise on body image. Although both exercises comprise training of muscular strength, balance, flexibility, and breathing (Liiv et al., 2013; McCall, 2013), yoga is much more focused on the individual itself than ballroom dance. Both types of exercise (yoga and ballroom dance) might require skills that train or are associated with interoception and proprioception (Schmalzl et al., 2015; Chauvigné et al., 2018) and training of interoception and proprioception both have been suggested to impact body image and body schema probably indirectly by changing neural network activity (Gandevia et al., 2002; Badoud and Tsakiris, 2017). Some results from previous studies propose that the focus on either of the two aspects is different in yoga compared to dancing: Whereas yoga promotes an internal focus of attention and observation in order to learn to align and synchronize breathing with yoga-related body postures (Schmalzl et al., 2015), dancing requires to align external stimuli (e.g., other dancers in the room, size of the room and floor itself) with one’s own body movements (Fonseca et al., 2014). Further possible factors that differ between yoga and ballroom dance in terms of body image could be related to social aspects (e.g., partnered dance, the role of music in dance), and notably—in combination with the different focus on interoception and proprioception—the perception of one’s own body in space (Fonseca et al., 2014; Lakes et al., 2016). These exercise-related differences should be examined further in future studies in order to understand their impact on body image. To further exploit the relevance of these factors, future studies could also incorporate other types of exercise such as gymnastics and, as in the current study, examine different facets of body image. As far as the present findings are concerned, no significant differences were found between the yoga group and the ballroom dance group, in neither of the self-report measures measuring perceptual, cognitive, or affective aspects related to body dissatisfaction and negative body image. As already mentioned, differences in age and gender between groups are unlikely to contribute to these results. Therefore, it seems that practitioners who report to preferentially practice ballroom dance or yoga on a regular basis might be comparable with regard to body dissatisfaction, body size distortion, and self-classified weight which needs to be examined in future studies. Of note, ballroom dance and yoga was also compared against team and individual sports groups (contrast 2). This combination of groups in contrast 2 was motivated by clinical studies that often combine yoga and dancing as body movement therapy in the treatment of body image in EDs. There were no differences observed for contrast 2.

### Different Types of Exercise and Well-Being

There also seems to be a relationship between overall exercising and mental health and well-being. The non-exercise group showed the highest mean depression scores in the PHQ-2 (Kroenke et al., 2003), screening for depressive symptoms, but also trait positive affect was significantly lower compared to the exercise groups. The findings conform to evidence from the literature that exercise of different forms (aerobic as well as anaerobic) is able to improve depression or mood (Reed and Ones, 2006; Kvam et al., 2016; Schuch et al., 2016) in comparison to not exercising. In the current study, all groups scored low in depressive symptoms on the PHQ-2 (Kroenke et al., 2003) and below the expected cut-off scores of clinical samples. Nevertheless, this already underpins previous studies, where the importance of carrying out exercises, because of their alleviating effect on mental health variables, was underlined (Penedo and Dahn, 2005; Mikkelsen et al., 2017). Specifically, the effects on well-being for the yoga and ballroom dance group found in this study need to be emphasized. Yoga practitioners showed the highest mean total physical activity per day. Whether yoga leads to a heightened awareness for health promotion and therefore to a more active lifestyle, or already active people prefer to carry out yoga, remains unclear. The ballroom dance group had the lowest scores on the PHQ-2 (Kroenke et al., 2003) and reported highest positive affect which supports previous studies on dance as therapeutic tool (Koch et al., 2014).

### Mediator Variable Influencing the Relationship Between Type of Exercise and Body Image

One factor that should be investigated further in the relationship between body image and exercise is physical efficacy. In the current study, it was tested as mediator between type of exercise contrasts and body dissatisfaction, body size distortion, and self-classified weight. Physical efficacy was found to be a mediator for the relation between type of exercise in contrast 1 (all exercise groups versus non-exercise group) and body image for all body image dimensions measured except for perceptual body size distortion assessed with the BIAS-BD (Gardner et al., 2009). It is important to note that in the mediator model the contrasts were used as exogenous/independent variable. The findings imply that physical efficacy is an important mediator explaining the difference in body image-related measures between all exercise groups and the non-exercise group. This difference between “exercising” (regardless of type of exercise) versus no exercise in body image would not have been found without physical efficacy in the model, suggesting that if differences in body image exist between exercising and not exercising, they can be detected only by including additional psychological factors such as physical efficacy.

In the present study, physical efficacy was measured via the physical efficacy scale from the FKKS questionnaire (Deusinger, 1998). The scale includes items such as “I am strong,” or “I perceive myself as pronouncedly stiff.” Thus, the items of the physical efficacy scale comprise attitudes of the individual that relate to the perceived and experienced degree of physical strength of the body related to motor skills and agility. There are certainly overlaps between the construct of physical efficacy with other concepts such as physical self-efficacy or physical functionality concerning the focus on the capability of the body instead of on appearance (Bandura, 1982; Alleva et al., 2015). Body functionality however not only includes perception of physical strength and motor skills as physical efficacy but also includes senses, body communication, and self-care, and it can therefore be seen as a broader concept than physical efficacy. Physical self-efficacy as part of Bandura’s social-cognitive theory (Bandura, 1977) is defined as the belief in one’s competence to complete a certain task in varying contexts (Bandura, 1982) and therefore often contains a subjective evaluation. Physical efficacy depicts a more general view of the body and its ability in an exercise setting and has a less task-oriented focus. Therefore, other than physical efficacy, physical self-efficacy and body functionality encompass certain perceptions and behaviors targeted at a certain aim, e.g., to carry out certain activities (Bandura, 1982; Alleva et al., 2015; Alleva et al., 2016). Concerning physical self-efficacy, there already exist findings that an improvement in physical self-efficacy could be associated with more physical activity being carried out (e.g., Peers et al., 2020). Alleva et al. (2016) found that a focus on body functionality served as a buffer protecting body image against negative consequences of viewing thin models depicted on social media and was associated with a higher body appreciation. What needs to be addressed further in future studies and which was not investigated in the context of the present study is which types of exercise or other activities are associated or lead to a higher physical efficacy. This might help to build interventions based on physical efficacy to alleviate negative body image. To this end, physical efficacy should also be examined in future studies with regard to positive body image.

### Limitations and Future Research

This study supports past research in the field of sports and exercise inasmuch as practicing exercises such as yoga, ballroom dance, team sports, or individual sports seems to be associated with a lower negative body image in terms of body dissatisfaction (perceptual, cognitive, and affective) in comparison to individuals who report to engage in no regular exercise. Moreover, this overall difference between exercise groups and the non-exercise group was mediated by physical efficacy. The results therefore shed further light on the question of whether practicing certain types of exercise influences body image differently and which psychological factors may mediate the difference in body image between exercise and no exercise. Nevertheless, the present survey study has several limitations that have to be taken into account and that need to be further explored in future studies. Although several dimensions of body image were considered in the current study, it has to be stated that no difference was made in body image perception between the social body and the sporting body (de Bruin et al., 2011). Body perception in everyday life might differ from the one experienced in the sporting environment. While a comparison to the average body in daily life might be associated with a positive body image, a comparison to other athletes’ bodies might be related to a negative body image with an athlete striving for a more muscular or thinner body shape (de Bruin et al., 2011). This leads to another limitation. Due to the recruitment in ballroom dance schools it can be expected that ballroom dancers did their exercise in a non-competitive way as normally competitions are mainly held by dance sports clubs but not schools. Nevertheless, it cannot be ruled out completely that not only in the ballroom dance group but also in the team and individual exercise groups, participation in any competitions might have taken place which can have an impact on body image. A comparison of these subgroups would therefore have been interesting with regard to body image. In this context, other factors like duration, frequency, or length of exercise should also be taken into account. In this study, there was only a difference found for duration of exercise. This variable was not considered in the contrast analyses because it was only retrieved from the exercise groups but not from the non-exercise group. Nevertheless, this variable could influence the results. Methodologically, the statistical procedure choosing four orthogonal contrasts diminishes the risk of a multiple testing bias. We complied with the approach by Zhao et al. (2010) concerning mediation analysis and interpretation by focusing on the indirect and direct effect rather than on all associations between variables. Although the total and specifically the direct effect of the contrast to the body image variable with the mediator in the model often had a different sign compared to the indirect effect, Zhao et al. (2010) conclude that in fact a mediation occurred, but there might be other omitted mediators that have to be found and taken into account. This underpins the necessity of finding and examining other possible psychological mediators. All in all, the results have to be interpreted with caution because they are only of correlational nature. A causal interpretation is not possible but warranted, in order to get more insight into how different types of exercise could improve body image. Only then, possible interventions can be constructed, especially in order to help people with a pronounced negative body image. A final limitation is, that, due to the assessment in a questionnaire format, only subjective self-report measures of the variables of interest were assessed. Sensitive variables like weight in order to compute the BMI are best measured objectively which requires either special scanning software or the assessment in a laboratory. On the other hand, online questionnaires often lead to a higher sample size. In order to evaluate the minimum effect size, a sensitivity analysis was conducted (Gpower; Faul et al., 2009). This revealed, for the present study, a minimum effect size of 0.2024 for the sample size of *N* = 270 (alpha level of 0.05, power of 0.80, according to Cohen, 1988).

## Conclusion

This study replicates findings of the importance of exercising for well-being and negative body image. Moreover, it goes beyond replication. It also offers new and promising research directions by comparing four different exercise groups that so far have not been studied together in a cross-sectional study. In this regard, particularly ballroom dance should be examined further differentiating it from other types of exercise. In order to get a better understanding of possible differences between types of exercise, underlying mechanisms should be explored further. In the present study, physical efficacy was found to be one important factor for the relationship between body image and type of exercise when contrasts were used in the mediation analysis. Therefore, the construct of physical efficacy should be investigated in future studies also with respect to its distinction to similar constructs. This brings us one step closer to understanding whether certain types of exercise are more advisable than others in fostering a positive body image and decreasing a negative body image. On top of that, possible interventions targeting the enhancement of physical efficacy, e.g., through exercising or other activities, could be developed and tested. This could facilitate the approach for many people to change the evaluation of their body, not only in everyday life but also in a clinical setting, and could be used in the treatment of diseases that are accompanied by body image concerns.

## Data Availability Statement

The data supporting the conclusions of this article will be made available by the authors, without undue reservation, to any qualified researcher. Due to the informed consent form in which the possibility of raw data being published online was not explicitly stated, only goup-level data, as it is provided in this manuscript, can be made accessible upon request.

## Ethics Statement

This study involving human participants was approved by the Local Ethics Committee of Ulm University, Germany. Participation in this study was voluntary and participants provided informed consent to participate in this study.

## Author Contributions

CH and VM did the conceptualization, methodology, validation, investigation, data curation, validation, writing—original draft, and writing—review, revision, and editing. VM did the visualization, and formal analysis. CH did the resources, supervision, project administration, and funding. Both authors contributed to the article and approved the submitted version.

## Conflict of Interest

The authors declare that the research was conducted in the absence of any commercial or financial relationships that could be construed as a potential conflict of interest.

## Footnotes

1 https://www.uni-ulm.de/einrichtungen/ethikkommission-der-universitaet-ulm/

2 Additional measures assessed in this survey were *Balanced Inventory of Desirable Responding* (BIDR; Paulhus, 1994), *Figural Muscle Scale* (developed by Prof. Cornelia Herbert^©^), *Multidimensional Assessment of Interoceptive Awareness* (MAIA; Mehling et al., 2012), *Reasons for Exercise Inventory* (Silberstein et al., 1988), *Stress- and Coping-Inventory* (SCI; Satow, 2012; German version), *Reasons for Exercise Inventory* (REI; Silberstein et al., 1988); *Twenty Statement Test* (TST; Kuhn and McPartland, 1954), and the *Toronto Alexithymia Scale* (TAS-20; Bagby et al., 1994). The Figural Muscle Scale by Herbert is a scale comprising athletic body silhouettes to allow particularly athletes to estimate their body in terms of size and shape in a realistic manner. The MAIA (Mehling et al., 2012), TAS (Bagby et al., 1994), and SCI (Satow, 2012) were included in the present survey to answer a second research question not directly related to the research question reported in this manuscript. The results of these questionnaires will be reported in a separate manuscript in reference to the study sample of the current manuscript to avoid any bias in publications.

## References

[B1] Alleva J. M. Martijn C. Van Breukelen G. J. Jansen A. Karos K. (2015). Expand your horizon: a programme that improves body image and reduces self-objectification by training women to focus on body functionality. *Body Image* 15 81–89. 10.1016/j.bodyim.2015.07.001 26280376

[B2] Alleva J. M. Veldhuis J. Martijn C. (2016). A pilot study investigating whether focusing on body functionality can protect women from the potential negative effects of viewing thin-ideal media images. *Body Image* 17 10–13. 10.1016/j.bodyim.2016.01.007 26878220

[B3] Ariel-Donges A. H. Gordon E. L. Bauman V. Perri M. G. (2019). Does yoga help college-aged women with body-image dissatisfaction feel better about their bodies? *Sex Roles* 80 41–51. 10.1007/s11199-018-0917-5

[B4] Badoud D. Tsakiris M. (2017). From the body’s viscera to the body’s image: is there a link between interoception and body image concerns? *Neurosci. Biobehav. Rev.* 77 237–246. 10.1016/j.neubiorev.2017.03.017 28377099

[B5] Bagby R. M. Parker J. D. A. Taylor G. J. (1994). The twenty-item Toronto Alexithymia scale-I: item selection and cross-validation of the factor structure. *J. Psychosom. Res.* 38 23–32. 10.1016/0022-3999(94)90005-1 8126686

[B6] Bandura A. (1977). Self-efficacy: toward a unifying theory of behavioral change. *Psychol. Rev.* 84 191–215. 10.1037/0033-295X.84.2.191 847061

[B7] Bandura A. (1982). Self-efficacy mechanism in human agency. *Am. J. Appl. Psychol.* 37 122–147. 10.1037/0003-066X.37.2.122

[B8] Benjamini Y. Hochberg Y. (1995). Controlling the false discovery rate: a practical and powerful approach to multiple testing. *J. R. Statist. Soc. B.* 57 289–300. 10.1111/j.2517-6161.1995.tb02031.x

[B9] Blair S. N. Morris J. N. (2009). Healthy hearts and the universal benefits of being physically active: physical activity and health. *Ann. Epidemiol.* 19 253–256. 10.1016/j.annepidem.2009.01.019 19344864

[B10] Booth F. W. Roberts C. K. Thyfault J. P. Ruegsegger G. N. Toedebusch R. G. (2017). Role of inactivity on chronic diseases: evolutionary insight and pathophysiological mechanisms. *Physiol. Rev.* 97 1351–1402. 10.1152/physrev.00019.2016 28814614 PMC6347102

[B11] Budde H. Schwarz R. Velasques B. Ribeiro P. Holzweg M. Machado S. (2016). The need for differentiating between exercise, physical activity, and training. *Autoimmun. Rev.* 15 110–111. 10.1016/j.autrev.2015.09.004 26384527

[B12] Campbell A. Hausenblas H. A. (2009). Effects of exercise interventions on body image: a meta-analysis. *J. Health Psychol.* 14 780–793. 10.1177/1359105309338977 19687115

[B13] Cash T. Winstead B. Janda J. (1986). Body image survey report: the great American shape-up. *Psychol. Today* 24 30–37.

[B14] Cash T. F. (2004). Body image: past, present, and future. *Body Image* 1 1–5. 10.1016/S1740-1445(03)00011-1 18089136

[B15] Cash T. F. (2015). “Multidimensional body-self relations questionnaire (MBSRQ),” in *Encyclopedia of Feeding and Eating Disorders*, ed. Wade T. (Singapore: Springer), 1–4. 10.1007/978-981-287-087-2

[B16] Caspersen C. J. Powell K. E. Christenson G. M. (1985). Physical activity, exercise, and physical fitness: definitions and distinctions for health-related research. *Public Health Rep.* 100 126–131. 3920711 PMC1424733

[B17] Chauvigné L. A. Belyk M. Brown S. (2018). Taking two to tango: fMRI analysis of improvised joint action with physical contact. *PLoS One* 13:e0191098. 10.1371/journal.pone.0191098 29324862 PMC5764359

[B18] Cohen J. (1988). *Statistical Power Analysis for the Behavioral Sciences*, 2nd Edn. Hillsdale, NJ: Erlbaum.

[B19] Cohen-Stratyner B. (2020). *Ballroom Dance.* Available online at: https://www.britannica.com/art/ballroom-dance (accessed August 29, 2020).

[B20] Cornelissen V. A. Fagard R. H. (2005). Effects of endurance training on blood pressure, blood pressure-regulating mechanisms, and cardiovascular risk factors. *J. Hypertens.* 46 667–675. 10.1161/01.HYP.0000184225.05629.51 16157788

[B21] Cruz C. J. G. D. Molina G. E. Porto L. G. G. Junquerira L. F. Jr. (2017). Resting bradycardia, enhanced postexercise heart rate recovery and cardiorespiratory fitness in recreational ballroom dancers. *Res. Q. Exercise Sport* 88 371–376. 10.1080/02701367.2017.1318202 28506112

[B22] de Bruin A. P. Oudejans R. R. D. Bakker F. C. Woertman L. (2011). Contextual body image and athletes’ disordered eating: the contribution of athletic body image to disordered eating in high performance women athletes. *Eur. Eat. Disord. Rev.* 19 201–215. 10.1002/erv.1112 21584913

[B23] Demarest J. Allen R. (2000). Body image: gender, ethnic, and age differences. *J. Soc. Psychol.* 140 465–472. 10.1080/00224540009600485 10981375

[B24] Deusinger I. M. (1998). *Die Frankfurter Körperkonzeptskalen (FKKS).* Göttingen: Hogrefe Verlag.

[B25] Du Y. Liu B. Sun Y. Snetselaar L. G. Wallace R. B. Bao W. (2019). Trends in adherence to the physical activity guidelines for Americans for aerobic activity and time spent on sedentary behavior among US adults, 2007 to 2016. *JAMA Netw. Open* 2:e197597. 10.1001/jamanetworkopen.2019.7597 31348504 PMC6661709

[B26] Dyremyhr ÅE. Diaz E. Meland E. (2014). How adolescent subjective health and satisfaction with weight and body shape are related to participation in sports. *J. Environ. Public Health* 2014:851932. 10.1155/2014/851932 25013414 PMC4074947

[B27] Fagard R. (2006). Exercise is good for your blood pressure: effects of endurance training and resistance training. *Clin. Exp. Pharmacol. Physiol.* 33 853–856. 10.1111/j.14401681.2006.04453.x 16922820

[B28] Faul F. Erdfelder E. Buchner A. Lang A.-G. (2009). Statistical power analyses using G^∗^Power 3.1: tests for correlation and regression analyses. *Beh. Res. Methods* 41 1149–1160. 10.3758/brm.41.4.1149 19897823

[B29] Field A. Miles J. Field Z. (2012). *Discovering Statistics Using R.* London: SAGE Publications.

[B30] Fong Yan A. Cobley S. Chan C. Pappas E. Nicholson L. L. Ward R. E. (2018). The effectiveness of dance interventions on physical health outcomes compared to other forms of physical activity: a systematic review and meta-analysis. *Sports Med.* 48 933–951. 10.1007/s40279-017-0853-5 29270864

[B31] Fonseca C. C. Thurm B. E. Vecchi R. L. Gama E. F. (2014). Ballroom dance and body size perception. *Percept. Mot. Ski.* 119 495–503. 10.2466/25.PMS.119c26z1 25349891

[B32] Forcier K. Stroud L. R. Papandonatos G. D. Hitsman B. Reiches M. Krishnamoorthy J. (2006). Links between physical fitness and cardiovascular reactivity and recovery to psychological stressors: a meta-analysis. *Health Psychol.* 25 1–36. 10.1037/0278-6133.25.6.723 17100501

[B33] Gandevia S. C. Refshauge K. M. Collins D. F. (2002). “Proprioception: peripheral inputs and perceptual interactions,” in *Sensorimotor Control of Movement and Posture: Advances in Experimental Medicine and Biology*, Vol. 158 eds Gandevia S. C. Proske U. Stuart D. G. (Boston, MA: Springer), 10.1007/978-1-4615-0713-0_8 12171152

[B34] Gardner R. M. Boice R. (2004). A computer program for measuring body size distortion and body dissatisfaction. *Behav. Res. Methods* 36 89–95. 10.3758/BF03195553 15190703

[B35] Gardner R. M. Jappe L. M. Gardner L. (2009). Development and validation of a new figural drawing scale for body-image assessment: the BIAS-BD. *J. Clin. Psychol.* 65 113–122. 10.1002/jclp.20526 19051276

[B36] Garner D. M. (1991). *Eating Disorder Inventory-2: Professional Manual.* Odessa, FL: Psychological Assessment Ressources.

[B37] Gomes da Silva Borges E. Cader S. A. Gomes, de Souza Vale R. Cruz T. H. P. de Gurgel de Alencar Carvalho M. C. (2012). The effect of ballroom dance on balance and functional autonomy among the isolated elderly. *Arch. Gerontol. Geriatr.* 55 492–496. 10.1016/j.archger.2011.09.004 22483371

[B38] Grogan S. (2017). *Body Image: Understanding Body Dissatisfaction in Men, Women and Children*, 3rd Edn. Oxon: Taylor & Francis.

[B39] Hackney M. E. Earhart G. M. (2010). Effects of dance on gait and balance in Parkinson’s disease: a comparison of partnered and nonpartnered dance movement. *Neurorehabil. Neural Repair* 24 384–392. 10.1177/1545968309353329 20008820 PMC2900796

[B40] Hausenblas H. A. Fallon E. A. (2006). Exercise and body image: a meta-analysis. *Psychol. Health* 21 33–47. 10.1080/14768320500105270

[B41] Hayes A. F. (2018). *Introduction to Mediation, Moderation, and Conditional Process Analysis: a Regression-based Approach*, 2nd Edn. New York, NY: The Guilford Press.

[B42] Hill J. Sandford R. Enright E. (2016). ‘It has really amazed me what my body can now do’: boundary work and the construction of a body-positive dance community. *Sport Soc.* 19 667–679. 10.1080/17430437.2015.1073946

[B43] Johnson F. Wardle J. (2005). Dietary restraint, body dissatisfaction, and psychological distress: a prospective analysis. *J. Abnorm. Psychol.* 114 119–125. 10.1037/0021-843X.114.1.119 15709818

[B44] Koch S. Kunz T. Lykou S. Cruz R. (2014). Effects of dance movement therapy and dance on health-related psychological outcomes: a meta-analysis. *Arts Psychother.* 41 46–64. 10.1016/j.aip.2013.10.004 PMC6710484 31481910

[B45] Koch S. C. Morlinghaus K. Fuchs T. (2007). The joy dance: specific effects of a single dance intervention on psychiatric patients with depression. *Arts Psychother.* 34 340–349. 10.1016/j.aip.2007.07.001

[B46] Kroenke K. Spitzer R. L. Williams J. B. W. (2003). The Patient Health Questionnaire-2: validity of a two-item depression screener. *Med. Care* 41 1284–1292. 10.1097/01.MLR.0000093487.78664.3C 14583691

[B47] Krohne H. W. Egloff B. Kohlmann C.-W. Tausch A. (1996). Untersuchungen mit einer deutschen Version der “Positive and Negative Affect Schedule” (PANAS). *Diagnostica* 42 139–156.

[B48] Kuhn M. H. McPartland T. S. (1954). An empirical investigation of self-attitudes. *Am. Sociol. Rev.* 19 68–76. 10.2307/2088175

[B49] Kunkel D. Robison J. Fitton C. Hulbert S. Roberts L. Wiles R. (2017). It takes two: the influence of dance partners on the perceived enjoyment and benefits during participation in partnered ballroom dance classes for people with Parkinson’s. *Disabil. Rehabil.* 8288 1–10. 10.1080/09638288.2017.1323029 28482703

[B50] Kvam S. Lykkedrang C. Hilde I. Hovland A. (2016). Exercise as a treatment for depression: a meta-analysis. *J. Affect. Disord.* 202 67–86. 10.1016/j.jad.2016.03.063 27253219

[B51] Lakes K. D. Marvin S. Rowley J. San Nicolas M. Arastoo S. Viray L. (2016). Dancer perceptions of the cognitive, social, emotional, and physical benefits of modern styles of partnered dancing. *Complement. Ther. Med.* 26 117–122. 10.1016/j.ctim.2016.03.007 27261991 PMC5267615

[B52] Lauche R. Sibbritt D. Ostermann T. Fuller N. R. Adams J. Cramer H. (2017). Associations between yoga/meditation use, body satisfaction, and weight management methods: results of a national cross-sectional survey of 8009 Australian women. *Nutrition* 34 58–64. 10.1016/j.nut.2016.09.007 28063513

[B53] Liiv H. Jürimäe T. Klonova A. Cicchella A. (2013). Performance and recovery: stress profiles in professional ballroom dancers. *Med. Probl. Perform. Art.* 28 65–69. 10.21091/mppa.2013.2012 23752279

[B54] Mahlo L. Tiggemann M. (2016). Yoga and positive body image: a test of the Embodiment Model. *Body Image* 18 135–142. 10.1016/j.bodyim.2016.06.008 27434106

[B55] McArdle W. D. Katch F. I. Katch V. L. (2006). *Essentials of Exercise Physiology.* Philadelphia, PA: Lippincott Williams & Wilkins.

[B56] McCall M. C. (2013). How might yoga work? An overview of potential underlying mechanisms. *J. Yoga Phys. Ther.* 3:1.

[B57] Mehling W. E. Price C. Daubenmier J. J. Acree M. Bartmess E. Stewart A. (2012). The multidimensional assessment of interoceptive awareness (MAIA). *PLoS One* 7:e0208034. 10.1371/journal.pone.0048230 23133619 PMC3486814

[B58] Mikkelsen K. Stojanovska L. Polenakovic M. Bosevski M. Apostolopoulos V. (2017). Exercise and mental health. *Maturitas* 106 48–56. 10.1016/j.maturitas.2017.09.003 29150166

[B59] Morano M. Colella D. Capranica L. (2011). Body image, perceived and actual physical abilities in normal-weight and overweight boys involved in individual and team sports. *J. Sports Sci.* 29 355–362. 10.1080/02640414.2010.530678 21184344

[B60] Neumark-Sztainer D. MacLehose R. F. Watts A. W. Pacanowski C. R. Eisenberg M. E. (2018). Yoga and body image: findings from a large population-based study of young adults. *Body Image* 24 69–75. 10.1016/j.bodyim.2017.12.003 29288970 PMC5869146

[B61] Olivardia R. Pope H. G. J. Borowiecki J. J. I. III Cohane G. H. (2004). Biceps and body image: the relationship between muscularity and self-esteem, depression, and eating disorder symptoms. *Psychol. Men Masc.* 5 112–120. 10.1037/1524-9220.5.2.112

[B62] Paulhus D. L. (1994). *Balanced Inventory of Desirable Responding: Reference Manual for BIDR Version 6.* Unpublished manuscript. Vancouver, BC: University of British Columbia.

[B63] Peers C. Issartel J. Behan S. O’Connor N. Belton S. (2020). Movement competence: association with physical self-efficacy and physical activity. *Hum. Movement Sci.* 70 102582. 10.1016/j.humov.2020.102582 31957668

[B64] Penedo F. J. Dahn J. R. (2005). Exercise and well-being: a review of mental and physical health benefits associated with physical activity. *Curr. Opin. Psychiatry* 18 189–193. 10.1097/00001504-200503000-00013 16639173

[B65] Piran N. (2015). New possibilities in the prevention of eating disorders: the introduction of positive body image measures. *Body Image* 14 146–157. 10.1016/j.bodyim.2015.03.008 25886711

[B66] Pöhlmann K. Roth M. Brähler E. Joraschky P. (2014). Der Dresdner Körperbildfragebogen (DKB-35): validierung auf der Basis einer klinischen Stichprobe. The dresden body image inventory (DKB-35): validity in a clinical sample. *Psychother. Psych. Med.* 64 93–100. 10.1055/s-0033-1351276 23966276

[B67] Questback GmbH (2016). *EFS Survey, Version Herbst 2016.* Köln: Questback GmbH.

[B68] Reed J. Ones D. S. (2006). The effect of acute aerobic exercise on positive activated affect: a meta-analysis. *Psychol. Sport Exerc.* 7 477–514. 10.1016/j.psychsport.2005.11.003

[B69] R Studio Team (2019). *RStudio: Integrated Development for R.* Boston, MA: RStudio, Inc.

[B70] Rucker D. D. Preacher K. J. Tormala Z. L. Petty R. E. (2011). Mediation analysis in social psychology: current practices and new recommendations. *Soc. Personal. Psychol. Compass* 5 359–371. 10.1111/j.1751-9004.2011.00355.x

[B71] Sabiston C. M. Pila E. Vani M. Thogersen-Ntoumani C. (2019). Body image, physical activity, and sport: a scoping review. *Psychol. Sport Exerc.* 42 48–57. 10.1016/j.psychsport.2018.12.010

[B72] Salci L. E. Martin Ginis K. A. (2017). Acute effects of exercise on women with pre-existing body image concerns: a test of potential mediators. *Psychol. Sport Exerc.* 31 113–122. 10.1016/j.psychsport.2017.04.001

[B73] Satow L. (2012). “Stress- und coping-inventar (SCI) [PSYNDEX Tests-Nr. 9006508],” in *Elektronisches Testarchiv*, ed. Leibniz-Zentrum für Psychologische Information und Dokumentation (ZPID) (Trier: ZPID).

[B74] Saß H. Wittchen H.-U. Zaudig M. (1996). *Diagnostisches und Statistisches Manual psychischer Störungen DSM-IV.* Göttingen: Hogrefe Verlag.

[B75] Schmalzl L. Powers C. Henje Blom E. (2015). Neurophysiological and neurocognitive mechanisms underlying the effects of yoga-based practices: towards a comprehensive theoretical framework. *Front. Hum. Neurosci.* 9:235. 10.3389/fnhum.2015.00235 26005409 PMC4424840

[B76] Schuch F. B. Vancampfort D. Richards J. Rosenbaum S. Ward P. B. Stubbs B. (2016). J. *Psychiatr. Res.* 77 42–51. 10.1016/j.jpsychires.2016.02.023 26978184

[B77] Silberstein L. R. Striegel-Moore R. H. Timko C. Rodin J. (1988). Behavioral and psychological implications of body dissatisfaction: do men and women differ? *Sex Roles* 19 219–232. 10.1007/BF00290156

[B78] Solomon-Krakus S. Sabiston C. M. Brunet J. Castonguay A. L. Maximova K. Henderson M. (2017). Body image self-discrepancy and depressive symptoms among early adolescents. *J. Adolesc. Health* 60 38–43. 10.1016/j.jadohealth.2016.08.024 27793726

[B79] Swami V. Harris A. S. (2012). Dancing toward positive body image? Examining body-related constructs with ballet and contemporary dancers at different levels. *Am. J. Dance Ther.* 34 39–52. 10.1007/s10465-012-9129-7

[B80] Thiel A. Jacobi C. Horstmann S. Paul T. Nutzinger D. O. Schüssler G. (1997). A German version of the Eating Disorder Inventory EDI-2. *Psychother. Psychosom. Med. Psychol.* 47 365–376. 9411465

[B81] Thiel A. Paul T. (2006). Test-retest reliability of the eating disorder inventory 2. *J. Psychosom. Res.* 61 567–569. 10.1016/j.jpsychores.2006.02.015 17011367

[B82] Thiel P. P. (2007). *Der Dresdner Körperbildfragebogen: Entwicklung und Validierung eines mehrdimensionalen Fragebogens.* Ph.D. thesis, Medical Faculty of the Technical University Dresden: Germany.

[B83] Tiggemann M. (2015). Considerations of positive body image across various social identities and special populations. *Body Image* 14 168–176. 10.1016/j.bodyim.2015.03.002 25865460

[B84] Tihanyi B. T. Böõr P. Emanuelsen L. Köteles F. (2016). Mediators between yoga practice and psychological well-Being: mindfulness, body awareness and satisfaction with body image. *Eur. J. Ment. Health* 11 112. 10.5708/ejmh.11.2016.1-2.7

[B85] Tylka T. L. Wood-Barcalow N. L. (2015). The Body Appreciation Scale-2: item refinement and psychometric evaluation. *Body Image* 12 53–67. 10.1016/j.bodyim.2014.09.006 25462882

[B86] Vankova H. Holmerova I. Machacova K. Volicer L. Veleta P. Celko A. M. (2014). The effect of dance on depressive symptoms in nursing home residents. *J. Am. Med. Dir. Assoc.* 15 582–587. 10.1016/j.jamda.2014.04.013 24913212

[B87] Voelker D. Reel J. Greenleaf C. (2015). Weight status and body image perceptions in adolescents: current perspectives. *Adolesc. Health, Med. Ther.* 6 149–158. 10.2147/ahmt.s68344 26347007 PMC4554432

[B88] Vossbeck-Elsebusch A. N. Waldorf M. Legenbauer T. Bauer A. Cordes M. Vocks S. (2014). German version of the Multidimensional Body-Self Relations Questionnaire - Appearance Scales (MBSRQ-AS): confirmatory factor analysis and validation. *Body Image* 11 191–200. 10.1016/j.bodyim.2014.02.002 24958652

[B89] Watson D. Clark L. A. Tellegen A. (1988). Development and validation of brief measures of positive and negative affect: the PANAS scales. *J. Pers. Soc. Psychol.* 54 1063–1070. 10.1037/0022-3514.54.6.1063 3397865

[B90] World Health Organization [WHO] (2010). *Global Recommendations on Physical Activity for Health.* Geneva: World Health Organization. 26180873

[B91] World Health Organization [WHO] (2013). *Global Physical Activity Questionnaire (GPAQ): Der “STEPwise Approach” zur Surveillance von Risikofaktoren für chronische Krankheiten. 1-3.* Geneva: World Health Organization.

[B92] Zhao X. Lynch J. G. J. Chen Q. (2010). Reconsidering Baron and Kenny: myths and truths about mediation analysis. *J. Consum. Res.* 37 197–206. 10.1086/651257

